# Organic Carbon and Nitrogen Isoscapes of Reef Corals and Algal Symbionts: Relative Influences of Environmental Gradients and Heterotrophy

**DOI:** 10.3390/microorganisms8081221

**Published:** 2020-08-11

**Authors:** Takanori Fujii, Yasuaki Tanaka, Koh Maki, Nobue Saotome, Naoko Morimoto, Atsushi Watanabe, Toshihiro Miyajima

**Affiliations:** 1 Atmosphere and Ocean Research Institute, The University of Tokyo, Kashiwanoha 5-1-5, Kashiwa 277-8564, Japan; takanori.fujii1984@gmail.com (T.F.); tanaka.yask@gmail.com (Y.T.); ko-maki@yakult.co.jp (K.M.); saotome@aori.u-tokyo.ac.jp (N.S.); nmorimoto@aori.u-tokyo.ac.jp (N.M.); 2 School of Environment and Society, Tokyo Institute of Technology, O-okayama 2-12-1 W8-13, Meguro, Tokyo 152-8552, Japan; a-watanabe@spf.or.jp

**Keywords:** carbon isotopes, compound-specific isotope ratios of amino acids, hermatypic coral, heterotrophy, microhabitat, nitrogen isotopes, *Symbiodinium*, terrestrial nutrient input

## Abstract

The elemental (C/N) and stable isotopic (δ^13^C, δ^15^N) compositions and compound-specific δ^15^N values of amino acids (δ^15^N_AA_) were evaluated for coral holobionts as diagnostic tools to detect spatiotemporal environmental heterogeneity and its effects on coral health. Hermatypic coral samples of eight species were collected at 12 reef sites with differing levels of pollution stress. The C/N ratios, δ^13^C values, and δ^15^N values of coral tissues and endosymbiotic algae were determined for 193 coral holobionts, and the amino acid composition and δ^15^N_AA_ values of selected samples were analyzed. δ^15^N values were influenced most by pollution stress, while C/N ratios and δ^13^C values depended most strongly on species. The results imply that δ^13^C and δ^15^N values are useful indicators for distinguishing the ecological niches of sympatric coral species based on microhabitat preference and resource selectivity. Using δ^15^N_AA_ values, the trophic level (TL) of the examined coral samples was estimated to be 0.71 to 1.53, i.e., purely autotrophic to partially heterotrophic. Significant portions of the variation in bulk δ^15^N and δ^13^C values could be explained by the influence of heterotrophy. The TL of symbionts covaried with that of their hosts, implying that amino acids acquired through host heterotrophy are translocated to symbionts. Dependence on heterotrophy was stronger at polluted sites, indicating that the ecological role of corals changes in response to eutrophication.

## 1. Introduction

The metabolic performance and architecture of coral-reef ecosystems depend on the delivery, allocation, and recycling of essential nutrient elements such as nitrogen (N) and phosphorus (P). Hermatypic corals in well-lit shallow reefs rely primarily on photosynthesis by endosymbiotic dinoflagellates (zooxanthellae) for carbon and energy sources. The reef corals acquire essential nutrients mainly from seawater pools of dissolved inorganic N (DIN) and P (DIP) similarly to free-living primary producers [1,2,3,4]. However, because the availability of DIN and DIP is limited and the ratio of DIN/DIP is not optimal in many coral reef habitats, corals often utilize supplementary sources of N and P, including heterotrophic feeding on zooplankton, bacterioplankton [5,6,7], and detritus [8,9]; dinitrogen fixation by symbiotic cyanobacteria [10]; and uptake of dissolved organic N and P from seawater [11,12,13]. Corals are able to survive in oligotrophic reef environments using these diverse nutrient sources and the efficient recycling systems associated with symbiosis [14].

Coral reefs are among the ecosystems most strongly affected by environmental changes in terms of distribution and biodiversity at both the global and local scales [15,16,17]. Extreme seasonal temperatures, for example, often cause acute degradation of shallow-water coral reefs due to bleaching, resulting in the replacement of sensitive species by tolerant species [18]. Local anthropogenic impacts such as eutrophication, hypoxia, and siltation also affect the local abundance and species composition of corals and may cause replacement of the coral community with macroalgal habitat or even barren sand flats [19,20]. Multiple stressors may affect corals synergistically. Increasing evidence indicates that the availability and relative abundance of N and P affect the responses of reef corals to detrimental stressors, such as heat-induced bleaching, ocean acidification, and pathogen infection [21,22,23,24,25,26]. In contrast, the increased nutrients and energy available through heterotrophic feeding may help corals to tolerate and recover from damage caused by heat stress and ocean acidification [27,28,29,30]. Thus, to diagnose the susceptibility and tolerance of corals to these lethal stressors, a rapid screening technique for evaluating the nutritional status of corals is needed.

Geographical mapping of the stable isotopic and elemental compositions of specific sessile organisms and sediments (isoscapes) has recently been applied to evaluate environmental gradients and the responses of resident organisms to such gradients at local to regional scales [31,32,33]. One aim of this study was to examine the usefulness of the bulk elemental ratio of carbon (C) to N (C/N) and the bulk stable isotope ratios of C (δ^13^C) and N (δ^15^N) as indicators of the general nutrient and energy status of corals and their symbiotic algae at the reef to island scale. For this purpose, we compared these parameters for eight coral species collected from 12 sites around Ishigaki Island, where detailed information is available about the spatial heterogeneity of environmental parameters including hydrodynamics [34]; nutrient status [35,36]; and the distribution of benthic communities, including hermatypic corals and seagrasses [37,38,39,40].

Although the C/N ratio of coral polyps is generally around 7 [41], the C/N ratios of algal symbionts may reflect the availability of dissolved inorganic nitrogen (DIN) in the ambient seawater [42,43], and may be affected by storage of excess lipids and carbohydrates in algal cells under nutrient-limited conditions [44]. Lipids are also stored in host coral tissues [45], and the amount of lipid storage, and thus the C/N ratio, of host tissues change due to environmental conditions [46,47] and may be responsible for tolerance against bleaching [48,49].

The bulk δ^13^C values of marine primary producers are primarily determined by the δ^13^C value of the dissolved inorganic carbon (DIC) used as a substrate, the ability to use HCO_3_^−^ in addition to CO_2_ (carbon concentration mechanisms), and isotope fractionation during the uptake and fixation of CO_2_. The variability of substrate δ^13^C values, i.e., δ^13^C of seawater DIC, is relatively low in typical coral reef waters at around 0–2‰ [50,51,52]. Although many species of zooxanthellae have their own carbonic anhydrases that enable them to use HCO_3_^−^ [53], the availability of DIC to zooxanthellae in hospite, which determines the magnitude of isotope fractionation during carbon fixation, appears to be regulated mainly by the host coral [54,55,56]. The magnitude of isotope fractionation depends on the kinetic balance of photosynthetic reactions and becomes greater when the carbon fixation reaction rather than the uptake of DIC is the rate-limiting step. Thus, δ^13^C values can be a good indicator of the overall photosynthetic performance and energy status of corals. The δ^13^C value of the host coral tissue is similar to that of the symbiotic algae when the former depends on the latter for C. However, corals can utilize alternative C sources such as zooplankton and dissolved organic matter through heterotrophy, and the δ^13^C values of such alternative sources usually differ from those of symbiotic algal products. As a result, the δ^13^C of the host coral tissue can differ from that of the symbionts, and this difference in δ^13^C may reflect the degree of dependence on heterotrophic C sources of the coral holobiont [57].

The δ^15^N values of corals, on the other hand, reflect the δ^15^N value of DIN when they depend on DIN as the N source. Because the δ^15^N values of anthropogenic N sources such as ammonium (NH_4_^+^) in sewage and nitrate (NO_3_^−^) in polluted groundwater are often much higher than that of natural DIN in surface seawater and that of newly fixed N by cyanobacteria, elevated δ^15^N values found in corals can be used as an indicator of nitrogen pollution [58,59,60]. The δ^15^N values of corals may be lowered through isotope fractionation when resources other than nitrogen (e.g., light) limit coral growth [61]. In contrast, the δ^15^N values of corals are elevated when they feed on zooplankton as an additional C and N source, because zooplankton have higher δ^15^N values than its food sources, phytoplankton and DIN, due to trophic isotope enrichment [62] and the δ^15^N values of corals are even higher than those of zooplankton due to further trophic enrichment [63]. Thus, to use the δ^13^C and δ^15^N values of corals as indicators of their photosynthetic performance and DIN sources, respectively, we must quantitatively evaluate the relative contributions of autotrophy and heterotrophy to metabolic demand.

The second aim of this study was to examine the compound-specific δ^15^N values of individual amino acids as a tool for determining the relative contribution of heterotrophy to coral nutrition. In heterotrophic organisms, the δ^15^N values of amino acids are determined by both the δ^15^N values of amino acids contained in the diet and the isotopic fractionation that occurs during various transamination reactions [64]; individual amino acids may be more or less enriched in ^15^N compared to the same amino acids in the diet depending on metabolic turnover. Some amino acids (“trophic-AA”) such as glutamic acid show relatively strong enrichment of ^15^N, whereas others (“source-AA”) such as phenylalanine show very little enrichment [65,66]. Because the magnitude of ^15^N enrichment for certain amino acids is conserved among many marine invertebrates and fish, the trophic distance of a consumer from the basal primary producers (i.e., the trophic position) can be estimated from the difference in δ^15^N values between the “trophic” and “source” amino acids [67]. This estimation can be performed without knowledge of the species composition or δ^15^N values of the primary producers at the base of the food chain. Evaluating the dependence of coral holobionts on heterotrophic nutrition is operationally equivalent to determining the trophic position of the host corals. Using the δ^15^N values of amino acids in coral tissues, we aimed to determine quantitatively the degree to which corals depend on heterotrophy as an N source and examine the factors that control coral heterotrophy.

## 2. Materials and Methods

### 2.1. Field Sampling

**Figure 1:**
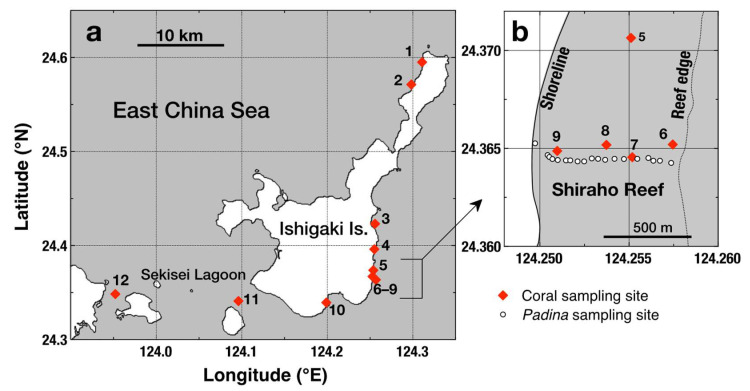
(**a**) Locations of sampling sites (solid diamonds) around Ishigaki Island, southwest Japan. Stations 5–9 are located in the well-studied Shiraho Reef fringing the east coast of the island. (**b**) A detailed map of Shiraho Reef stations. The open-circle points in (**b**) indicate sampling points of the macroalga *Padina* spp. collected in 2002 and used for comparison.

Coral samples were collected from 12 reef sites around Ishigaki Island in the northwestern Pacific Ocean (Figure 1). Stations 4 (near a river mouth), 9 (affected by submarine groundwater discharge), and 10 (near a sewage outfall) receive anthropogenic nutrient inputs [36]. River and groundwater discharge near Stations 4 and 9, respectively, contain around 300 µM NO_3_^−^, while the sewage effluent discharged near Station 10 often contains >500 µM NH_4_^+^ (as the freshwater endmember concentration). The other sites are affected by external nutrients to varying degrees, depending on distance from major point sources. Stations 11 and 12 (Sekisei Lagoon) are the least affected sites.

Coral sampling was conducted in four seasons, including August of 2009 and January, May, and August of 2010 (Table S1). Around 100 g (wet weight) of coral fragments were collected using a chisel or hand saw from large colonies. The collected species included *Acropora clathrata* Brook, 1891, *Acropora digitifera* Dana, 1846, *Acropora pulchra* Brook, 1891, *Acropora vaughani* Wells, 1954, *Favites chinensis* Verrill, 1866, *Heliopora coerulea* Pallas, 1766, *Porites cylindrica* Dana, 1846, and *Porites lutea* Quoy and Gaimard, 1833. All collected species are common in the study area. Samples were stored individually in plastic bags and placed in the dark for up to 1.5 h during transportation to the field laboratory (International Coral Reef Monitoring Center, Ishigaki Island). In the laboratory, corals were temporarily maintained in natural seawater with aeration.

Individual coral samples were placed into clean plastic bags (280 × 200 mm, 0.08 mm thick) with 30 mL of filtered seawater (FSW) prepared by filtering natural offshore seawater (<0.3 µM DIN, <0.03 µM DIP) through precombusted (450 °C, 3 h) glass fiber filters (Whatman GF/F, 47 mm; GE Healthcare, Chicago, IL, USA). Organic tissue of the coral was scraped from the carbonate skeleton using a precleaned stainless steel brush and collected in FSW. The suspension of coral tissue was transferred to a 50-mL polypropylene centrifuge tube and centrifuged at 2000× *g* for 6 min at room temperature. The supernatant was transferred to a new tube and centrifuged again under the same conditions. Centrifugation was repeated three times, and the final supernatant was transferred to a new plastic tube and stored at −25 °C until further treatment. This sample was thereafter considered “the host coral fraction” of the original coral holobiont. The pellet from the first centrifugation was resuspended in 30 mL of fresh FSW and kept still until the carbonate debris settled. The supernatant was decanted into a new plastic tube. The suspended particles were washed twice through centrifugation and resuspension in a similar manner to that described above. Appropriate aliquots of the final suspension were filtered through precombusted glass fiber filters (Whatman GF/F, 25 mm) to collect algal cells on them. These filter samples were referred to as “the algal symbiont (zooxanthella) fraction” of the holobiont and stored at −25 °C until further treatment. For a few selected coral samples, the mucus fraction was collected by rinsing the surface of colony fragments with chilled ultrapure water (Milli-Q, Merck-Millipore, Burlington, MA, USA), then the mixture of mucus and ultrapure water was packed into a 100-mL polypropylene bottle and frozen at −25 °C.

The frozen suspension of the host coral fraction and frozen mucus solution were freeze-dried, homogenized using a mortar and pestle, and the resulting powdered samples were stored in glass vials until analysis. The filter samples of symbiotic algae were freeze-dried and stored in a desiccator until analysis.

### 2.2. Bulk Stable Isotope Analysis

All samples were subjected to acid treatment prior to analysis to remove inorganic C. About 60 mg of homogenized powder of host coral tissue or mucus was placed in a silver container for elemental analysis (10 × 10 mm, SÄNTIS Analytical AG, Teufen, Switzerland), to which 100 µL of 1.0 N HCl was added. The container was not disturbed until bubbling of CO_2_ (if any) ceased. Then, the container was heated on a hotplate at 60 °C until the contents dried completely. The silver container was folded and packed into an elemental analysis grade tin container (10 × 10 mm, SÄNTIS Analytical AG, Teufen, Switzerland) in a small cubic shape. The filter sample containing the symbiotic algal fraction was placed in a disposable Petri dish and treated with HCl fumes for 48 h in a large airtight plastic box to remove inorganic C. Petri dishes containing the filter samples were then transferred to a vacuum desiccator that held a small amount of NaOH pellets as an acid absorber. The filters were kept in vacuo for 3 to 5 days to remove absorbed HCl and water. A few filters without samples were treated similarly and used for filter blank correction. Then, each filter in a tin container (10 × 10 mm, SANTIS Analytical) was formed into a small tablet using a hand-held tablet press.

Analysis was carried out using an elemental analyzer-isotopic ratio mass spectrometer (EA-IRMS) system (FLASH EA/Conflo III/DELTA plus XP, Thermo Scientific, Bremen, Germany) to determine the organic carbon (OC) and total nitrogen (TN) contents along with bulk C and N stable isotope ratios. The measured OC and TN contents were used to estimate the OC/TN (C/N) ratio of each sample. The C and N isotope ratios are reported using conventional δ-notation (δ^13^C, δ^15^N) with Vienna Pee Dee Belemnite (VPDB) and atmospheric N_2_ as reference standards, respectively:

(1) $$\delta^{m}E=\left(\frac{R_{s}}{R_{\mathrm{rs}}}-1\right)\times 1000\;(‰)$$

where *R*_s_ and *R*_rs_ are the isotope ratios, calculated as ^m^E/^m−1^E, of the sample and the reference standard, respectively, and m = 13 or 15 for E = C or N, respectively. Working standard materials (L-histidine (δ^13^C = −10.2‰; δ^15^N = −7.8‰), glycine (−33.8‰; +1.3‰), and L-alanine (−19.6‰; +10.1‰); SI-Science, Tokyo, Japan) were used for calibration. The reproducibility (1σ) of measurements of replicate reference samples (*n* = 4–6) for the C/N molar ratio, δ^13^C, and δ^15^N was within ±0.2, ±0.1‰, and ±0.2‰, respectively.

### 2.3. Amino Acid Composition

About 10 mg of freeze-dried powdered sample of host coral tissue or mucus, or a piece of dried glass fiber filter containing symbiotic algae, was treated in 6 N HCl at 110 °C for 12 h to hydrolyze the peptides into free amino acids. The hydrolysate was dried under a stream of N_2_ at 40 °C and then redissolved in an appropriate amount of ultrapure water (Milli-Q, Merck-Millipore, Burlington, MA, USA. This solution was injected into a high-performance liquid chromatography (HPLC) system (NANOSPACE SI-1, Shiseido, Tokyo, Japan) equipped with an autosampler (SIL-10, Shimadzu Scientific Instruments Inc., Kyoto, Japan), a semi-micro reverse-phase separation column (CAPCELL PAK C18, Shiseido, Tokyo, Japan), and a fluorometric detector, in which pre-column derivatization of amino acids with *o*-phthaldialdehyde [68] was performed automatically after injection. The following amino acids were quantified: alanine (Ala), glycine (Gly), valine (Val), leucine (Leu), isoleucine (Ile), aspartic acid + asparagine (Asx), threonine (Thr), β-alanine (β-Ala), serine (Ser), methionine (Met), glutamic acid + glutamine (Glx), phenylalanine (Phe), histidine (His), tyrosine (Tyr), arginine (Arg), ornithine, and lysine (Lys). Cysteine (Cys), proline (Pro), and tryptophan (Trp) could not be quantified using this method, and Met may also have been partially lost during the acid hydrolysis. The total amino acid concentration was defined as the sum of all amino acids detected. The amino acid composition is given as the mole percentage of each amino acid in total amino acids.

### 2.4. Compound-Specific δ^15^N Analysis of Amino Acids

Compound-specific N isotope analysis of amino acids was carried out after *N*-pivaloyl-isopropyl derivatization according to a previously reported [69] and modified [70] method. Briefly, an appropriate amount of dried sample (homogenized powder of the host coral and mucus fractions or dried glass fiber filter of the symbiotic algal fraction) was loaded into a Reacti-Vial (Thermo Fisher Scientific, Waltham, MA, USA) and hydrolyzed in concentrated HCl at 110 °C for 12 h. After cooling, the lipid fraction was removed through extraction with dichloromethane followed by hexane+dichloromethane (6 + 5). The hydrophilic fraction containing amino acids was dried under an N_2_ gas stream. Amino acids were first derivatized using isopropanol+thionyl chloride (4 + 1) and then using dichloromethane+pivaloyl chloride (1 + 1) to generate *N*-pivaloyl-isopropyl esters. The derivatives were extracted using hexane+dichloromethane, dried under an N_2_ gas stream, and finally dissolved in dichloromethane. The amounts of samples used were determined based on the results of bulk carbon and nitrogen analyses, described above, so that the amounts of representative amino acids such as Glx and Phe would be >0.2 µmol.

The amino acid solution was injected into a gas chromatography/combustion/isotope ratio mass spectrometry (GC/C/IRMS) system (Agilent GC6890/Combustion III/DELTA plus XP, Thermo Scientific, Bremen, Germany). An Ultra-2 capillary column (50 m × 0.32 mm; 0.52-µm film thickness; Agilent Technologies, Santa Clara, CA, USA) was used for separation in splitless mode. The temperatures of the injector, column oven, and combustion and reduction furnaces, as well as the gas flow rate were set according to a previous study [71]. For most samples, measurement was repeated three times, and the mean and standard deviation of δ^15^N (δ^15^N_AA_) values were calculated for each amino acid. Using this method, the δ^15^N values of the following amino acids were determined: Ala, Gly, Val, Leu, Ile, Pro, Asx, Thr, Ser, Met, Glx, Phe, and Tyr. However, we found that determination of the δ^15^N values of amino acids other than Val, Glx, and Phe was sometimes affected by poor chromatographic resolution of peaks due to overlap with other amino acid derivatives (e.g., Leu and Ile) or unidentified contaminants (e.g., Pro and Met). The reproducibility (1σ) of the determination of the δ^15^N values of Glx and Phe was usually within 1‰ and never exceeded 1.4‰, while that of other amino acids sometimes exceeded 1.5‰. Note that glutamine and asparagine lose their carbamoyl-N during acid hydrolysis, while the amino-N is left behind; they are therefore measured as glutamic acid and aspartic acid, respectively.

### 2.5. Data Treatment and Statistical Analysis

We analyzed elemental and bulk isotope data from a total of 139 coral samples for which reliable measurements of both the endosymbiont and host coral fractions were available. To determine the compound-specific δ^15^N values of amino acids, we used 18 datasets for the host coral fraction and 10 datasets for the symbiotic algal fraction. Through microscopic examination, we found that separation of algal cells from the host coral tissue with the method we used was sometimes incomplete, especially for samples of *Heliopora coerulea* and *Porites* spp. Slight contamination with host cells in the symbiotic algal fraction is unlikely to cause serious bias in elemental and bulk isotope analyses. However, such contamination might have caused significant bias in measurements of δ^15^N_AA_ values, as the difference in δ^15^N values of amino acids such as Glx between the host coral and algal symbionts can be large, especially when the host depends strongly on heterotrophy for its nutrition. Therefore, we avoided δ^15^N_AA_ data from certain algal samples for which significant contamination could not be excluded.

For statistical analyses, including analysis of variance (ANOVA) and multiple comparison analysis, and curve fitting, we used the software packages Aabel NG1 v. 4.20 (Gigawiz Ltd.Co., Tulsa, OK, USA and Pro Fit v. 7.0.14 (QuantumSoft, Uetikon am See, Switzerland). Details of these analyses are provided in the Results section.

## 3. Results

### 3.1. C/N Ratio and Bulk Carbon and Nitrogen Isotope Ratios

**Figure 2:**
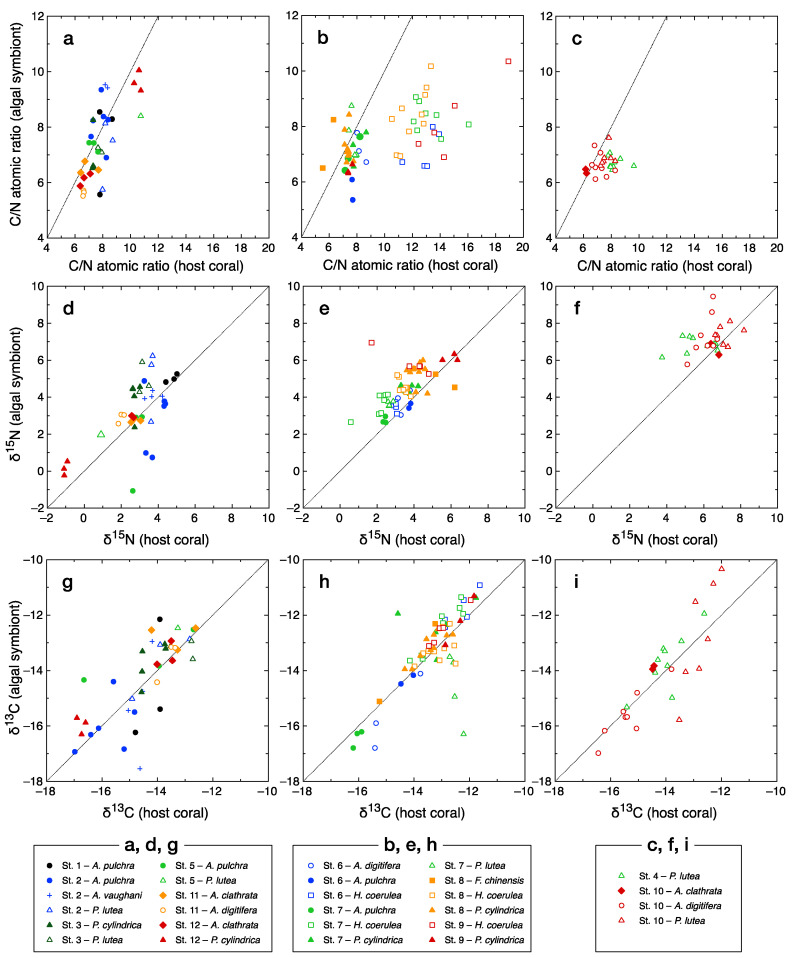
The C/N atomic ratio (**a**–**c**), bulk δ^15^N (**d**–**f**), and bulk δ^13^C values (**g**–**i**) of the host coral fraction (abscissa) and symbiotic algal (zooxanthella) fraction (ordinate) of coral samples. Samples in plots (**a**,**d**,**g**) were collected from reef sites relatively distant from pollution sources. Samples in plots (**b**,**e**,**h**) were collected from Shiraho Reef, which receives significant groundwater seepage that contains a high concentration of anthropogenic nitrate. Samples in plots (**c**,**f**,**i**) were collected from the most polluted sites, i.e., the river-mouth Station 4 and the sewage-affected Station 10.

The C/N atomic ratio of the symbiotic algal fraction of the coral samples (*n* = 139) ranged from 5.4 to 10.4, with the majority of the samples having values around 7 (Figure 2a–c). Aside from *Heliopora coerulea* and five samples of other species, the C/N ratio of the host coral fraction was similar to that of the symbiotic algal fraction from the same holobiont (difference within ± 2). The host coral fraction of *H. coerulea* had high C/N ratios of 10.5–18.9. Bulk δ^15^N varied widely from −1.1‰ to +9.5‰ (Figure 2d–f), with relatively high values recorded for samples collected at the river-mouth Station 4 and sewage-affected Station 10 (Figure 2f). The bulk δ^13^C values of host coral tissues and algal symbionts ranged between −17.5‰ and −10.3‰ (Figure 2g–i). Aside from three samples, the difference in δ^15^N or δ^13^C values between the host coral and the symbiotic algal fractions of the same holobiont was less than ±3‰; aside from 20 samples, the difference was within ±2‰ for both δ^15^N and δ^13^C.

Seasonal variations in the C/N ratios, bulk δ^15^N values, and bulk δ^13^C values of the host coral and symbiotic algal fractions were evaluated with one-way ANOVA and Bonferroni–Dunn post hoc analysis (α = 0.05) using seven subsets of data to compare multiple samples (*n* ≥ 3) of the same coral species collected from the same sampling station among seasons (Table S2). The seasonal difference in C/N ratios was significant only for the symbiotic algal fraction of *Porites lutea* collected from Station 4 and the host coral fraction of *Acropora pulchra* collected from Station 2. The seasonal difference in bulk δ^15^N values was significant for only one of seven comparisons of the symbiotic algal fraction, and three of seven comparisons of the host coral fraction, while the seasonality of bulk δ^13^C values was significant for two of seven and three of seven comparisons of the symbiotic algal and host coral fractions, respectively. In general, the seasonal differences were small and statistically insignificant. Where significant results were obtained, δ^15^N and δ^13^C values were lower in winter (January) than in summer (May and August).

Species-specific differences in the C/N ratio, δ^15^N, and δ^13^C were similarly evaluated using 14 subsets of data to compare two or more species in multiple samples collected at the same station during the same season (Table S3). The octocoral *Heliopora coerulea*, which occurred only at Stations 6, 7, 8, and 9, had significantly higher C/N ratios than corals belonging to other genera, especially in the host coral fraction. In contrast, *Acropora* spp. often had significantly lower C/N ratios than other genera. Significant differences in δ^15^N values were detected in some species combinations, although the order of species was not consistent. The difference in δ^13^C values in the symbiotic algal fraction was significant only in a few cases, with the δ^13^C values of the host coral fraction often being higher for *Porites lutea* than other species.

Spatial variations in the C/N ratio, δ^15^N, and δ^13^C were similarly evaluated using nine subsets of data to compare multiple samples of the same coral species collected at the same sampling event among stations (Table S4). Aside from a few cases, spatial differences in the C/N ratio and bulk δ^13^C values were not statistically significant. In contrast, the spatial difference in bulk δ^15^N values was significant for eight of nine comparisons in both the symbiotic algal and host coral fractions. In general, the δ^15^N values of samples collected from Stations 1, 8, 9, and 10 were relatively high, while those for samples from Stations 2, 3, 6, 7, 11, and 12 were relatively low.

**Figure 3:**
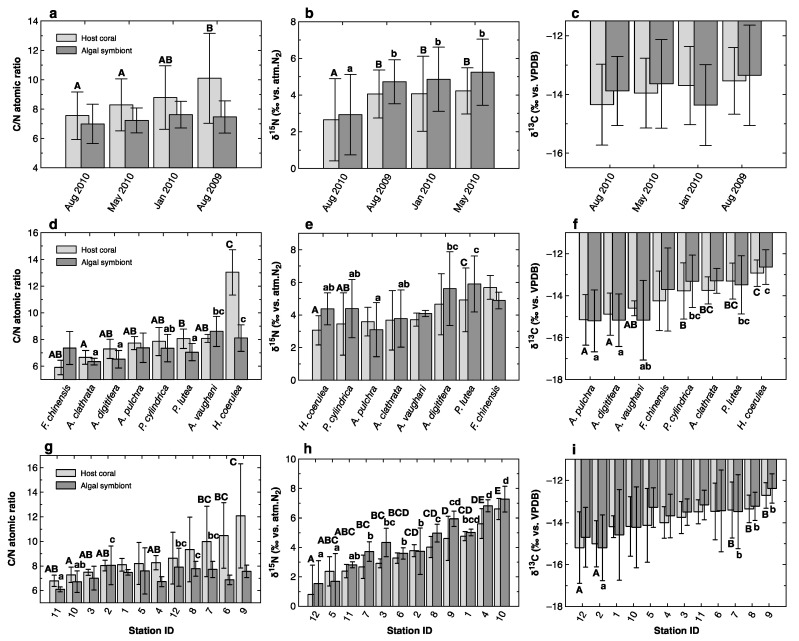
Comparison of average C/N ratios (**a**,**d**,**g**), δ^15^N values (**b**,**e**,**h**), and δ^13^C values (**c**,**f**,**i**) of host-coral (thin grey) and symbiotic algal (thick grey) fractions of coral samples among sampling events (**a**–**c**), species (**d**–**f**), and sampling sites (**g**–**i**). The data were averaged and arranged in increasing order based on host-coral fraction values in each plot. Error bars represent standard deviation. Differences among events, species, and sites were evaluated using the Tukey–Kramer multiple comparison test (α = 0.05), and different characters at the top of the error bars indicate statistically significant differences. Upper- and lower-case characters correspond to the host coral and symbiotic algal fractions, respectively. Lack of characters indicates that no significant difference was detected.

Differences in C/N ratio, δ^15^N, and δ^13^C between seasons were evaluated after combining all data (*n* = 139) for different species and stations (Table 1 and Figure 3a–c). In Figure 3 (and Figure S1), mean values are shown in ascending order based on the host coral fraction; differences between mean values were examined using the Tukey–Kramer multiple comparison test with α = 0.05. The C/N ratio of the host coral fraction was significantly lower in May and August 2010 than in August 2009, while the δ^15^N values of both the host coral and zooxanthella fractions were significantly lower in August 2010 than in other months. No seasonal difference was detected in δ^13^C values.

The species-specific C/N ratio, δ^15^N values, and δ^13^C values were also compared after combining all data across seasons and stations (Figure 3d–f). Inter-specific variations in C/N ratios and δ^15^N values were relatively small compared to the intra-specific (i.e., seasonal and spatial) variations represented by error bars, except for the extremely high C/N ratio recorded for the host coral fraction of *H. coerulea* (Figure 3d). In contrast, the δ^13^C values of three *Acropora* species (*A. pulchra*, *A. digitifera*, and *A. vaughani*) were significantly lower than those of *H. coerulea* and *Porites* spp.

The spatial variations in these parameters were evaluated after compiling data for all species and seasons (Figure 3g–i). The C/N ratios of the host coral fraction were relatively high at Stations 6, 7, 8, and 9 (Figure 3g) because *H. coerulea*, which has particularly high C/N ratios (Figure 3d), occurred only at these four stations (Table S1). The C/N ratios of the symbiotic algal fractions did not follow the same spatial patterns as those of the host coral fractions. Spatial differences were best resolved using bulk δ^15^N values (Figure 3h). The δ^15^N values of the host coral fraction were significantly higher at river-mouth Station 4, groundwater-influenced Station 9, and sewage-affected Station 10, relative to Stations 3, 5, 7, 11, and 12, where terrestrial loading was apparently low. The δ^15^N values of the algal fractions showed a similar trend, apparently reflecting the same environmental gradient. The δ^13^C values of the host coral and symbiotic algal fractions were significantly higher in samples from Stations 7, 8, and 9 than in those from Station 2 (and Station 12 for the host coral fraction) (Figure 3i), corresponding to the finding that *H. coerulea*, which had relatively high δ^13^C values (Figure 3f), occurred exclusively at Stations 6–9.

The differences in C/N ratios between the symbiotic algal and host coral fractions (ΔC/N_a–h_) were particularly large for *H. coerulea* (−4.9 ± 1.6, mean ± 1σ; Figure S1d). Excluding this species, ΔC/N_a–h_ values did not differ significantly (−0.56 ± 0.90) among species. ΔC/N_a–h_ values were significantly lower in August 2009 compared to other months (Figure S1a), and relatively low at Stations 6, 7, and 9 (Figure S1g). The difference in bulk δ^15^N values between the symbiotic algal and host coral fractions (Δδ^15^N_a–h_) of *H. coerulea* were highest (1.3 ± 1.0) and were significantly higher than those of *A. pulchra* (−0.5 ± 1.3) (Figure S1e). Excluding these species, little species-, site-, or season-specific variation was observed in Δδ^15^N_a–h_ values (Figure S1b,e,h). In contrast, differences in bulk δ^13^C values between the algal and host coral fractions (Δδ^13^C_a–h_) depended strongly on season (*p* < 0.0001, ANOVA), with the lowest mean value obtained in winter (January 2010; Figure S1c). No significant difference in Δδ^13^C_a–h_ values was detected among species or sites (Figure S1f,i).

In summary, spatial variation was most evident for bulk δ^15^N in both the symbiotic algal and host coral fractions. Season and species had relatively small effects on δ^15^N values. In contrast, C/N ratios, especially those of the host coral fraction, were largely determined by species. Differences in C/N ratios and δ^15^N values between host corals and algal symbionts appeared to be species-specific and did not show significant dependence on site or season. Bulk δ^13^C values varied mainly with species, while site- and season-specific differences in δ^13^C values were relatively small. The difference in δ^13^C values between host corals and algal symbionts depended solely on season.

### 3.2. Amino Acid Composition and Compound-Specific δ^15^N Values of Amino Acids

The amino acid compositions of the host coral and symbiotic algal fractions were determined for 10 selected coral samples (*Acropora digitifera* (1), *A. pulchra* (3), *A. vaughani* (1), *Heliopora coerulea* (2), *Porites cylindrica* (2), and *P. lutea* (1)). The most abundant amino acid was Glx (23.9 ± 5.9 mol-% in the host coral fraction, 18.0 ± 3.7 mol-% in the symbiotic algal fraction; mean ± SD), followed by Asx (12.3 ± 3.2%, 12.1 ± 1.3%), and Gly (10.7 ± 1.7%, 12.0 ± 1.3%), while the abundance of Phe was 3.8 ± 1.3% and 3.7 ± 0.7% in the host coral and symbiotic algal fractions, respectively (Table S5). The composition was similar among all species tested, although *P. cylindrica* contained relatively high levels of Glx (32–36% in the host coral, and 23–26% in the algal symbionts). Note that Pro was not included in the analysis because it is undetectable by the HPLC method we used. The ratio of amino acid-N to total N varied among samples from 56 to 80%. Although the amino acid composition we observed is very similar to the results reported by Fitzgerald and Szmant [72], the differences in relative abundance among amino acids in our results are larger than theirs, reflecting differences in the sample preparation method (Table S5).

**Figure 4:**
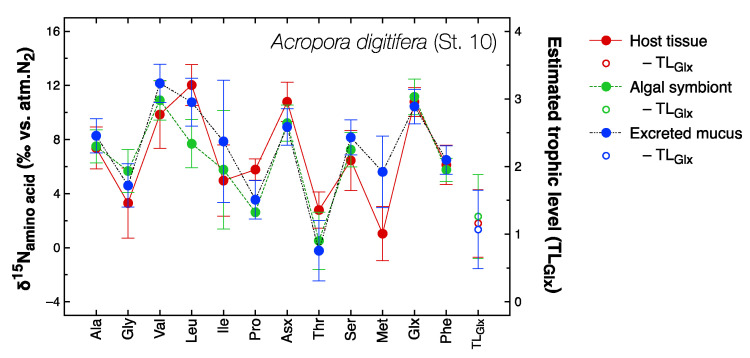
Compound-specific δ^15^N values of amino acids extracted from the host tissue (open symbols), algal symbiont (solid symbols), and mucus (cross) fractions of *Acropora digitifera* collected from Station 10. TL_Glx_ is the trophic position estimated from the difference in δ^15^N values between Glx and Phe using Equation (2) [67]. Error bars represent the standard deviation of triplicate analyses of the same sample. The δ^15^N values of Met could not be determined for the symbiotic algal fraction due to insufficient sample quantity.

The compound-specific δ^15^N values of amino acids were determined for 18 selected host coral samples and 10 symbiotic algal samples. The overall pattern of δ^15^N values among 12 amino acids was similar in all samples tested and is illustrated in Figure 4 for *Acropora digitifera*. In general, Glx, Asx, and Val had relatively high δ^15^N values compared to Gly, Thr, and Phe. Because measurement of Glx and Phe had good chromatographic resolution and provided fairly reliable δ^15^N values, we estimated the apparent trophic level (TL_Glx_) of corals using these two values and the following equation [64]:

(2) $${\mathrm{TL}}_{\mathrm{Glx}}=\frac{\delta^{15}N_{\mathrm{Glx}}-\delta^{15}N_{\mathrm{Phe}}-3.4}{7.6}+1$$

where δ^15^N_Glx_ and δ^15^N_Phe_ represent the δ^15^N of Glx and Phe, respectively, measured in the same sample. The value 3.4 represents the mean difference of δ^15^N_Glx_ from δ^15^N_Phe_ in marine microalgae, and 7.6 is the mean difference in trophic enrichment of δ^15^N_Glx_ relative to that of δ^15^N_Phe_ in marine invertebrates and fish [64]. For example, TL_Glx_ values calculated for the host coral and symbiotic algal fractions extracted from, as well as coral mucus excreted by, the same colony of *A. digitifera* (Figure 4) were very similar at about 1.0, i.e., the trophic level of a primary producer.

**Figure 5:**
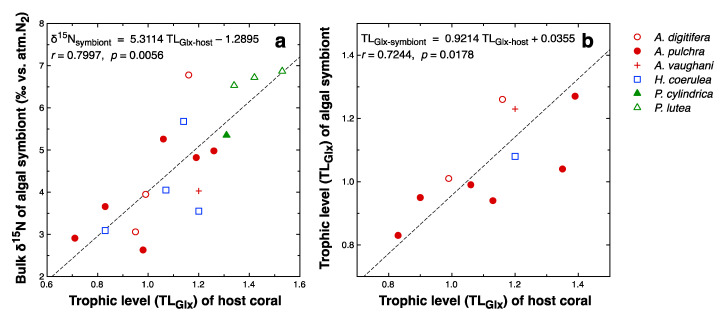
The bulk δ^15^N values (**a**) and estimated trophic position TL_Glx_ (**b**) of the symbiotic algal fractions plotted against the TL_Glx_ values of the host tissue fractions of the same coral holobionts. Linear regression results are provided.

The δ^15^N_Glx_ and δ^15^N_Phe_ values of the host coral fractions ranged from 5.2 to 13.0‰ and 1.5 to 6.4‰, respectively. Those of the symbiotic algal fractions varied from 4.6 to 11.2‰ and 1.2 to 5.8‰, respectively. δ^15^N_Glx_ values were closely correlated with bulk δ^15^N in both the host coral (*r* = 0.8973; slope = 1.18) and algal symbiont (*r* = 0.7843; slope = 1.29) fractions. TL_Glx_ values of the host coral and symbiotic algal fractions ranged from 0.71 to 1.53 and 0.83 to 1.27, respectively. The standard deviation of TL_Glx_ values of the same samples based on triplicate estimates was 0.01–0.24. The TL_Glx_ values of the host coral fractions were positively correlated with the bulk δ^15^N values of the algal symbionts (*r* = 0.7997, *p* = 0.0056; Figure 5a) and those of the host corals (*r* = 0.7386, *p* = 0.0093). The TL_Glx_ values of the algal symbionts were not always unity, but instead covaried significantly with the TL_Glx_ values of the host corals (*r* = 0.7244, *p* = 0.0178; Figure 5b).

## 4. Discussion

### 4.1. Seasonal Variations

As the study sites are under subtropical climate conditions, they experience significant seasonal changes in air and water temperature, solar radiation, wind regime, and precipitation [12,73]. The monthly average air temperature is highest in July (ca. 30 °C) and lowest in January (ca. 19 °C). Precipitation occurs throughout the year (100–300 mm month^−1^) and is relatively intense (>200 mm month^−1^) during the Intertropical Convergence Zone (ITCZ)-affected season (May and June) and typhoon season (August to October). Atmospheric deposition of nitrogen is relatively extensive during the winter months (January to March) due to the northwestern monsoon, which conveys atmospheric pollutants from continental China [74]. Therefore, corals may show significant seasonality in their metabolic activities and elemental and isotopic compositions.

In a few cases, the δ^13^C and δ^15^N values of the host coral tissues and symbiotic algae (zooxanthellae) of the coral holobionts were significantly higher in summer (May and August) than in winter (January) (Table S2) due to the relatively low metabolic activity of the corals in winter. When the photosynthetic and assimilation rates are reduced due to low irradiation or low temperature, the effect of isotope fractionation during assimilation of DIC and DIN into organic molecules becomes more apparent, resulting in lower (or more negative) δ^13^C and δ^15^N values in newly produced biomass [59]. The more negative δ^13^C values in algal symbionts relative to their host coral fractions (i.e., negative Δδ^13^C_a–h_) observed in winter (Figure S1c) can be explained by the same mechanism. Because the metabolic turnover rate of algal symbionts is higher than that of host coral tissues [14], the δ^13^C and δ^15^N values of the algae respond more quickly to changes in water temperature and light intensity compared to those of the host coral. As a result, Δδ^13^C_a–h_ values become negative when metabolic activity is reduced in winter and rise with increasing metabolic activity in summer (Figure S1c).

However, the seasonal variations in C/N, δ^15^N, and δ^13^C values were relatively small and often overwhelmed by species-specific and spatial variations. For example, seasonal differences are apparent in the C/N ratios of the host coral fractions and ΔC/N_a–h_ values (Figure 3a, Figure S1a, and Table 1), but this is because the samples collected in August 2009 contained abundant *Heliopora coerulea* (Table S1), for which the C/N ratios of the host coral fractions were high (Figure 3d). After removing the data for *H. coerulea*, C/N ratios and ΔC/N_a–h_ values show no significant seasonal variation (*p* > 0.26 and 0.51 based on ANOVA, respectively). To evaluate precisely the small seasonal variability in δ^15^N, δ^13^C values, and especially in C/N ratios, more intensive and carefully designed sampling is required. For a first approximation, we ignore seasonal variability and consider the pooled dataset from all sampling events in the following discussion.

### 4.2. Species-Specific Differences

The elemental and isotopic compositions of corals reflect the specific metabolic and structural characteristics of individual species. Therefore, consistent differences among different species in C/N ratios, δ^15^N values, and δ^13^C values are expected. However, few studies to date have systematically investigated the species-specific differences in these parameters.

For example, microhabitat preference differs greatly among species. Water circulation in the vicinity of coral colonies can vary strongly both within and among sites depending on the rate and direction of prevalent tidal flows and microtopography [34]. Some species such as *Acropora* spp. prefer, and may occur exclusively in, exposed habitats with high water circulation rates. Other species such as *Porites* and *Heliopora* prefer or tolerate more sheltered habitats, where mass transfer of essential nutrients tends to be limited. In the latter type of environment, uptake of DIC and DIN by coral holobionts is often limited by diffusion, and consequently, the δ^13^C and δ^15^N values of corals in such habitats are generally higher than those of corals living in exposed habitats (cf. [75,76] for the same phenomenon in benthic algae). Dependence on heterotrophic nutrition also differs among species, and the isotopic ratios of corals that depend heavily on feeding are influenced by those of their prey. As zooplankton, a common food source for corals, generally have lower δ^13^C and higher δ^15^N values than corals in the same habitats, corals are expected to show increasingly lower δ^13^C values and higher δ^15^N values as they consume more zooplankton.

A species-specific difference in δ^13^C values was evident in both the host coral and symbiotic algal fractions (Figure 3f and Table 1). The δ^13^C values of both fractions were significantly higher in *Heliopora coerulea* than in *Acropora pulchra*, *A. digitifera*, and *A. vaughani*, and also significantly higher in *Porites lutea* and *P. cylindrica* than in *A. pulchra* and *A. digitifera*. Meanwhile, no significant difference was found in Δδ^13^C_a–h_ values among species (Figure S1f).

A possible explanation for the differences in δ^13^C values may be differing lipid contents in the sampled tissues because lipids usually have lower δ^13^C values than other organic components of tissues [57,77]. The lipid contents of the corals were not determined in this study. However, if the observed low δ^13^C values were a result of high lipid content, a negative correlation would have been observed between δ^13^C values and C/N ratios, as the latter would have been elevated in samples with higher lipid contents [78]. This was not the case. For example, *H. coerulea* showed the highest mean δ^13^C values and the highest C/N ratios (Figure 3d,f). *Acropora pulchra* and *A. digitifera*, whose δ^13^C values were lowest, had relatively low C/N ratios. Moreover, as most lipids are stored in host coral tissues rather than their algal symbionts [45], the effect of lipids on δ^13^C values would be strongest in the host coral fraction. In our analysis, the host coral and symbiotic algal fractions showed almost identical variations in δ^13^C (Figure 3f). Thus, the variations in δ^13^C among species do not appear to be related to differences in lipid content.

A more plausible explanation is that microhabitat preference differs among species, as noted above. *Acropora* spp. generally prefer more exposed, high-turbulence microhabitats. The strong water motion typical of such habitats reduces the diffusion boundary layer thickness and relieves corals from diffusion limitation in DIC uptake, which is prevalent in more sheltered, low-turbulence microhabitats. As a result, isotope fractionation in C fixation reactions is more evident and the δ^13^C values of the tissues produced are more negative in these species (Figure 3f). In addition, the branched morphology typical of *Acropora* may play a role in reducing the diffusion boundary layer around them [79], resulting in relief from diffusion limitation of DIC and DIN uptake and lower δ^13^C and δ^15^N values in their organic tissues. In contrast, significantly higher δ^13^C and δ^15^N values are often found for *P. lutea* (Table S3), perhaps due in part to its massive form, which makes the holobionts more susceptible to diffusion limitation.

One of the most remarkable species-specific characteristics observed in this study was the very high C/N ratios of the host-coral fractions from *Heliopora coerulea*, which contained almost double the amount of organic C per unit N compared to the symbiotic algal fractions from the same holobionts and compared to the host and symbiont fractions of other species (Figure 3d). Thus, it is plausible that *H. coerulea* accumulates C-rich substances in the host tissue to a greater degree than do other species. Because the δ^13^C values of the host coral fractions of *H. coerulea* were relatively high, carbohydrates, which have δ^13^C values similar to or higher than other biomass components [77,80], likely constitute the major C storage compounds in this species. This species tolerates heat stress and has higher survival rates than other species during extensive bleaching events [37]. Its high C storage capacity may be responsible for these physiological characteristics.

Excluding *H. coerulea*, the variability in C/N ratios was slightly higher for the symbiotic algal fractions than the host coral fractions, and this effect was especially strong at oligotrophic stations (Figure 2a) relative to mesotrophic (Figure 2b) and eutrophic (Figure 2c) stations. Tanaka et al. [43] reported that corals experimentally enriched with N had lower C/N ratios than non-enriched corals, and that this difference was greater in the endosymbionts than in the host tissues. Although no such consistent trend was observed in our data, nutrient limitation at the oligotrophic stations may have resulted in excess production and temporary storage of organic C in the algal symbionts of some coral holobionts, with differences among species and microenvironments, which in turn led to relatively high variability in symbiotic algal C/N ratios at these stations.

Significant differences among species were also detected in δ^15^N values (Figure 3e). Interpretation of these differences is difficult because, as discussed below, δ^15^N values showed clear spatial patterns (Figure 3h) and some of the observed species-specific differences in δ^15^N values can be explained by differences in the spatial distributions of species. At Stations 7, 8, and 9, the δ^15^N values of both the host coral and the symbiotic algal fractions of *P. cylindrica* were consistently significantly higher than those of *H. coerulea* (Table S3). This difference implies that these two species use different sources of N. Specifically, *P. cylindrica* appears to depend on heterotrophic nutrition to a greater degree than does *H. coerulea*. In contrast, at Station 12 the same species, *P. cylindrica,* showed characteristically low δ^15^N and δ^13^C (Figure 2d,g) values, which may indicate strong dependence on dinitrogen fixation to support the N demand of these holobionts at this oligotrophic site [10]. Thus, both δ^13^C and δ^15^N values are useful indicators for distinguishing the ecological niches of sympatric coral species based on microhabitat preference and N resource selectivity.

### 4.3. Spatial Variations

The δ^15^N values of the host coral and symbiotic algal fractions showed remarkable spatial variations (Figure 3h and Table S4). δ^15^N values were particularly high in samples collected from the sewage-influenced Station 10, the river-mouth Station 4, and the groundwater-influenced Station 9, likely reflecting uptake of anthropogenic N inputs by corals. River water collected near Station 4 and groundwater collected from wells near Station 9 contained high concentrations of NO_3_^−^ (120–380 µM) with high δ^15^N values (6.2–8.8‰; Table 2). In addition, sewage water flowing into Station 10 contained very high levels of NH_4_^+^ (110–550 µM) with extremely high levels of δ^15^N (21.2‰; Table 2). On the other hand, corals collected at sites relatively distant from anthropogenic pollution sources (e.g., Stations 11 and 12) had δ^15^N values of −1.1 to +3.1‰ in both the host coral and symbiotic algal fractions (Figure 2d), which may be considered the baseline range of δ^15^N values without local pollution. Particulate organic matter contained in the offshore water of Ishigaki Island and NO_3_^−^ contained in meteoric water collected on the island showed similar ranges of δ^15^N (Miyajima et al., unpublished data). Thus, the δ^15^N values of corals can be used as an index of the relative contribution of local-pollution N sources to meeting the N demand of corals [36], while the site-specific average δ^15^N values (Figure 3h) represent the degree of N pollution input at each site. 

**Figure 6:**
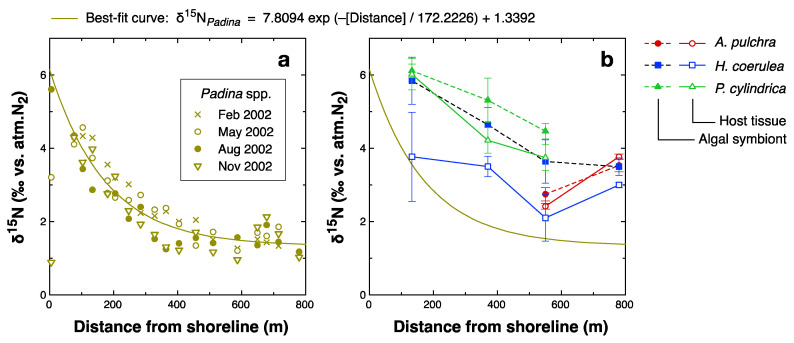
Spatial distribution of bulk δ^15^N values across the reef flat of Shiraho Reef (Figure 1b). (**a**): δ^15^N values of the macroalgae *Padina* spp. collected in 2002 (redrawn from [84]). Sampling points are shown in Figure 1b (open circles). Solid lines in (**a**,**b**) are best-fit exponential curves for macroalgal δ^15^N values. (**b**): δ^15^N values of coral samples measured in this study. Sampling points from left to right are Stations 9, 8, 7, and 6. Error bar represents standard deviation.

A spatial trend of decreasing δ^15^N values with increasing distance from the shoreline has been reported previously for the macroalgae *Padina* spp., which was collected at several sites around Ishigaki Island [36]. Due to seepage of groundwater with high NO_3_^−^ concentrations, which was frequently observed along the shoreline of their study sites, Umezawa et al. [36] proposed that the spatial distribution of macroalgal δ^15^N values was caused by the spatial gradient of the availability of groundwater NO_3_^−^, which decreased from emission sources along the shoreline to the offshore area. The detailed spatiotemporal distribution of δ^15^N of *Padina* spp. determined later at Shiraho Reef (Figure 6a) [84], had a pattern of consistent decrease from the shoreline to the reef crest (ca. 800 m from the shoreline), with negligible seasonal changes. Although the year of sample collection differed, coral samples collected at Stations 6–9 in this study had a similar spatial pattern of δ^15^N values to *Padina* (Figure 6b). This finding supports the possibility that the spatial trend in the δ^15^N values of the coral samples from Stations 6–9 resulted from differential groundwater NO_3_^−^ inputs. An offset of δ^15^N was also found between the coral samples and *Padina* spp. (Figure 6b). In particular, the δ^15^N values of *Porites cylindrica* were consistently higher than those of *Padina,* by 2–4‰. This difference may be due in part to differences between the sampling years; that is, the δ^15^N values of both natural and anthropogenic DIN sources for this reef may have been higher in 2009–10 than in 2002 for unknown reasons. However, we have no direct evidence or rationale at present to support such interannual differences in DIN sources. Alternatively, the offset of δ^15^N may be related to corals being partially dependent on heterotrophic nutrition to meet their N demand. Although we did not collect δ^15^N data for zooplankton in the study area, zooplankton often has significantly higher δ^15^N values than phytoplankton due to trophic enrichment. Thus, corals that use zooplankton as their food source would have higher δ^15^N values relative to primary producers, including *Padina*, at the same site. The possible heterotrophy of the corals studied here is discussed further in the next section.

Differences in C/N ratios and δ^13^C values among sampling stations were relatively minor and often insignificant (Table S4 and Figure 3). The C/N ratios (Figure 3g) and δ^13^C values (Figure 3i) of the host coral fractions were relatively high and ΔC/N_a–h_ values (Figure S1g) were more negative at Stations 6–9 relative to other stations. These differences are likely related to *H. coerulea*, which had the highest average C/N ratios and δ^13^C values and the most negative ΔC/N_a–h_ values among the coral species studied (Figure 3d,f, and Figure S1d), and occurred only at Stations 6–9 (Table S1). Therefore, we cannot conclude that there are any consistent trends in C/N ratios or δ^13^C values among stations that are independent of species-specific differences.

### 4.4. Contribution of Heterotrophic Nutrition

Evaluating the relative contributions of heterotrophic and autotrophic nutrition in coral holobionts is operationally equivalent to determining the trophic position of the host corals. Bulk δ^13^C vs. δ^15^N mapping has been established as a standard method for identifying the trophic position of consumers in a given food web [62,85]. However, several issues complicate application of this method to trophic analysis of animals with symbiotic plants, as the method relies on the assumption that the animals depend solely on food as a source of both C and N. In the case of symbiotic organisms such as zooxanthellate corals, the animal partner depends on primary production by its symbiotic algae for both C and N. The symbiotic algae (zooxanthellae) can rapidly translocate photosynthates to the animal (host coral) within several minutes of production for use as precursor molecules in biomass synthesis [86,87]. Thus, the δ^13^C and δ^15^N values of the symbiont and host may be almost identical, as both build their biomass from the same pool of precursors. However, it has been suggested that a host may occasionally digest the symbiotic partners or their products [14,88,89]. In this case, the δ^13^C and δ^15^N values of the host would be somewhat higher than those of their symbionts depending on the trophic isotope enrichment specific to that host. Furthermore, host corals can capture external food sources such as zooplankton and assimilate C and N heterotrophically [6]. In this case, the δ^13^C and δ^15^N values of the host are affected by those of its food sources, and the strength of this effect depends on the relative importance of heterotrophy to its nutrition. The relative importance of heterotrophy to the host may differ between C and N. For example, a host coral could acquire C mainly from its algal symbionts and N through heterotrophy. Moreover, the effects on the δ^13^C and δ^15^N values of the algal symbionts from food consumed by the host remain unclear. All of these factors complicate interpretation of bulk δ^13^C–δ^15^N distributions in trophic analysis of symbiotic systems such as coral holobionts.

Compound-specific N isotope analysis of amino acids (δ^15^N_AA_) has recently been applied as a new and precise tool for determining the trophic position of animals in the food web. One merit of this method is that it can estimate trophic positions based on the δ^15^N_AA_ value of the animal alone, without knowledge of the isotopic composition of its food sources or primary producers at the base of the food web [67]. Thus, most of the difficulties in determining the trophic positions of symbiotic organisms through the bulk isotope method may be resolved by using the new δ^15^N_AA_ method. However, studies of the δ^15^N_AA_ in the trophic analysis of symbiotic systems are still very few [90].

Two assumptions must be satisfied for application of this method to a given food web. First, the δ^15^N_AA_ values of individual amino acids in primary producers must follow a consistent pattern. In other words, the δ^15^N values of various amino acids relative to a reference amino acid (e.g., Glx) should be similar across all primary producers in the food web. Second, the trophic enrichment factor of δ^15^N_AA_ should be defined individually for each amino acid. The magnitude of trophic enrichment correlates with the relative metabolic turnover time of each amino acid and has positive values for some amino acids (“trophic-AA”) and zero or very small values for other amino acids (“source-AA”) [66]. Theoretically, the trophic position of an organism in the food web can be estimated under these assumptions based on the δ^15^N values of at least one trophic-AA and one source-AA [67]. First, we examine whether these assumptions are met by coral holobionts.

We estimated the fractionation coefficients of individual amino acids with respect to glutamic acid (Glx) for the symbiotic algal fraction of the corals (*n* = 10) and compared them with typical coefficients for eukaryotic algae and cyanobacteria, as summarized by McCarthy et al. [91] (Table 3). The fractionation coefficient ε is conventionally defined as

(3) $$\varepsilon =\left(\frac{R_{x}}{R_{\mathrm{Glx}}}-1\right)\times 1000\;(‰)$$

where *R* = ^15^N/^14^N, and x is an amino acid other than Glx. *R* is related to δ^15^N as shown in Equation (1). The standard deviation in estimates of ε from 10 algal samples was small (<2‰), except for those of Pro and Thr. McCarthy et al. [91] classified amino acids into four “non-fractionating” and eight “fractionating” compounds, defined by ε having a value close to zero or a relatively large negative value (ε < −2), respectively. This classification appears to fit the results for symbiotic algae, except that Leu had an ε value close to zero, and thus might be included in the “non-fractionating” group (Table 3). Comparison of these data implies that the patterns of the δ^15^N_AA_ values of the symbiotic algae are similar to the typical patterns of the eukaryotic algae studied by McCarthy et al. [91], and therefore symbiotic algae are amenable to trophic analysis based on δ^15^N_AA_.

Trophic enrichment of δ^15^N_AA_ in corals was examined using the host coral fraction of holobionts (*n* = 18). The δ^15^N_AA_ value of a consumer can be modeled as depending on both the consumer’s trophic position and the δ^15^N_AA_ values of primary producers at the base of the food web:

(4) $$\delta^{15}N_{x,\mathrm{consumer}}=\left(\mathrm{TL}-1\right)\times \Delta_{x}+\delta^{15}N_{x,\mathrm{primary}\;\mathrm{producer}}$$

where x is an amino acid, TL is the trophic level of the consumer, and Δ_x_ is the trophic enrichment factor for δ^15^N_x_. However, the δ^15^N value of phenylalanine (δ^15^N_Phe_) is insensitive to trophic position and instead directly reflects the δ^15^N_Phe_ value of the diet or the basal food resource (i.e., Δ_Phe_ ~ 0; [70]). The δ^15^N_Phe_ values of the host coral fractions varied from 1.4‰ to 6.5‰. Thus, we defined Δδ^15^N_x–Phe_ as:

(5) $$\Delta \delta^{15}N_{x-\mathrm{Phe}}=\delta^{15}N_{x}-\delta^{15}N_{\mathrm{Phe}}$$

where x is an amino acid other than Phe. Δδ^15^N_x–Phe_ can be assumed to depend solely on trophic position as long as the first assumption is met. Then, we classified amino acids based on the correlation of Δδ^15^N_x–Phe_ with Δδ^15^N_Glx–Phe_ (Table 4). Because Glx is regarded as a typical “trophic-AA” whose δ^15^N value depends strongly on trophic position [70], other amino acids may be classified into the “trophic-AA” or “source-AA” groups when their Δδ^15^N_x–Phe_ values covary with Δδ^15^N_Glx–Phe_ values or do not, respectively. According to this criterion, Val, Leu, and Asx, for which the correlation was strong (*r* > 0.8, *p* < 0.01; Table 4), are classified as trophic-AA, while Thr and Met showed no correlation and thus are classified as source-AA along with Phe. Ala, Gly, Ile, and Ser showed weak yet significant correlations and may be included in trophic-AA. The classification of Pro is questionable because the measurement of δ^15^N_Pro_ was not sufficiently reliable, as explained above. Overall, the classification described here aligns well with that originally determined for marine plankton [66]. Therefore, we tentatively conclude that the trophic analysis method using δ^15^N_AA_ values originally proposed for marine pelagic food webs by McCarthy et al. [66] can be applied to determining the degree of heterotrophy in coral holobionts.

Whether the magnitude of trophic enrichment of δ^15^N_AA_ for each trophic-AA (i.e., Δ_x_ in Equation (4)) differs between corals and other free-living heterotrophs in marine food webs remains unclear. To quantify the trophic enrichment factor, changes in δ^15^N_AA_ must be monitored experimentally for coral holobionts growing heterotrophically. Such an experiment would be quite difficult, as most hermatypic corals obligately depend on photosynthesis by their algal symbionts. Here, we assumed that the trophic enrichment factors of coral holobionts for various δ^15^N_AA_ match those established for free-living marine consumers [67]. The dependence of coral holobionts on heterotrophic nutrition was evaluated using the reported average enrichment factors of marine heterotrophs for Glx and Phe. The trophic positions of the host coral and symbiotic algal fractions were evaluated using Equation (2), and dependence on heterotrophy was determined by whether TL_Glx_ exceeded one.

When the coral holobiont grows autotrophically, the host coral must either obtain amino acids from the symbiotic algae or synthesize them itself. The particular amino acids that the host coral can synthesize vary among host species [72,92,93]. However, host corals are generally capable of synthesizing glutamic acid, either from NH_4_^+^ and organic carbon translocated from the symbionts [94] or through transamination from glutamine translocated from the symbionts [95]. This general rule is supported by the finding of this study that trophic position (TL_Glx_), estimated from δ^15^N_Glx_ and δ^15^N_Phe_, is close to one for many host corals (Figure 5a). Thus, both the host coral and symbiotic algae appear to show a δ^15^N_AA_ pattern characteristic of primary producers (TL = 1) when the holobiont grows autotrophically. When the host coral consumes external food sources, TL_Glx_ may rise with increasing dependence on heterotrophy. Assuming that the trophic enrichment factor associated with heterotrophy in the host coral is similar to those of other heterotrophic organisms such as fish, and that the main food source of the corals is zooplankton with TL_Glx_ = 2, then the TL_Glx_ of the host coral should vary from 1 to 3 as the dependence of its nutrition on heterotrophy varies from 0 to 100%. Estimated TL_Glx_ for the host coral fraction was up to 1.53 (Figure 5a), indicating that the corals studied here depended on heterotrophy to meet up to 27% of their N demand.

In this study, the TL_Glx_ values of the symbiotic algal fractions varied in accordance with those of the host coral fractions (Figure 5b). It has conventionally been assumed that the coral holobiont recycles N internally so that the catabolic product NH_4_^+^ generated by the host coral is reused by the symbionts to synthesize new amino acids [14,96]. In such a case, the TL_Glx_ of the algal symbionts must always be one. The positive correlation of TL_Glx_ between the host and symbiont fractions (Figure 5b) implies instead that a portion of the amino acids acquired by the host coral through feeding is shared with the symbionts for use in protein biosynthesis. In other words, the translocation of amino acids between the host and symbionts of a holobiont appears to be bilateral, rather than solely benefitting the host. Experiments have demonstrated that host corals can translocate to their endosymbiotic algal cells a portion of amino acids that the hosts absorbed as dissolved free amino acids from the external medium [97] or acquired through feeding of brine shrimps [98]. The translocation of heterotrophically acquired amino acids implied by the results of this study likely relies on a similar mechanism. Through recycling and translocation, N can be shared and retained within the symbiosis with a long turnover time [14].

Another result of note is the positive correlation between the TL_Glx_ values of host corals and the bulk δ^15^N values of algal symbionts (Figure 5a). This finding has two implications. First, the observed variability in the bulk δ^15^N values of the algal symbionts may be attributed not only to the δ^15^N of DIN sources but also, to some degree, on heterotrophy of the host corals. The maximum extent to which feeding on zooplankton by host corals affects the bulk δ^15^N and bulk δ^13^C values of the holobiont cannot be clearly determined from our data. Although the δ^15^N and δ^13^C values of zooplankton at the study site are unknown, our unpublished data (suspended particles collected near Station 5 at night when the zooplankton density was very high) imply that the δ^15^N and δ^13^C values of zooplankton are likely around 8‰ and −22‰, respectively. Assuming that the baseline δ^15^N and δ^13^C values of the holobiont without heterotrophic nutrition are 2‰ and −13‰, respectively (Figure 2), that the holobiont acquires a maximum of 27% of its N and C from feeding on zooplankton, and that the trophic enrichment factors of bulk δ^15^N and δ^13^C for corals are 3.3‰ and 0.4‰, respectively [85], then the δ^15^N and δ^13^C values of the holobiont will rise by a maximum of 2.5‰ and fall by a maximum of 2.3‰, respectively, from their baseline values due to heterotrophy. These ranges are large relative to the ranges of variation in bulk δ^15^N and δ^13^C values (Figure 2). For example, the differences in bulk δ^15^N values observed between macroalgae and corals at Stations 6–9 (Figure 6b) were generally within this range, and thus may be explained by species-specific effects of heterotrophy. When using δ^15^N and δ^13^C values of corals to infer the contributions of specific N and C sources through isotope mass balancing, the effect of heterotrophy on these parameters must be taken into account, for example, by using the δ^15^N_AA_ method.

The second implication of the positive correlation between bulk δ^15^N and TL_Glx_ values (Figure 5a) is that the dependence of the coral holobiont on heterotrophy may increase under eutrophic conditions. Because the bulk δ^15^N values of algal symbionts were largely determined by the δ^15^N of the available DIN, and the latter was strongly constrained by eutrophication caused by pollutant DIN with high δ^15^N values, the correlation indicates that heterotrophy is generally high in corals growing in polluted environments. This relationship may be explained as follows. Input of pollutant DIN stimulates primary production by phytoplankton and enhances the pelagic food chain relative to the benthic food chain. As corals can exploit resources through heterotrophy from the pelagic, rather than benthic, food chain, the dependence of corals on heterotrophy increases with increasing input of pollutant N. Thus, corals may act as an important agent of pelagic–benthic coupling in coastal ecosystems during the eutrophication process. This factor should be considered when modeling ecosystem responses to eutrophication.

Trophic analysis based on δ^15^N_AA_ has great potential for disentangling the complex trophic interactions present in symbiotic systems such as coral holobionts. However, few studies have yet applied compound-specific isotope analysis of important metabolites such as amino acids to trophic analysis of animal–plant symbiotic systems [90]. This study provides a frame of reference for interpreting δ^15^N_AA_ patterns found in coral holobionts and similar endosymbiotic associations in future advanced studies.

## 5. Conclusions

In this study, we explored the potential of bulk biomass properties such as C/N ratios and the stable isotope ratios δ^13^C and δ^15^N in the host coral tissue and symbiotic algal cells to be used for spatial and temporal analyses of the environmental and health conditions of coral holobionts. We demonstrated that the dependence of coral holobionts on heterotrophy can be successfully evaluated through trophic analysis based on compound-specific δ^15^N values for individual amino acids. The main results can be summarized as follows:

1. C/N ratios were species-specific and were not very sensitive to changes in environmental conditions. However, excess organic C production under nutrient-limited conditions may be reflected in higher C/N ratios of algal endosymbionts.
2. δ^13^C values appeared to be driven by overall isotope fractionation during DIC uptake and fixation related to the hydrodynamic conditions of the microhabitat and coral morphology, which constrain the thickness of the diffusion boundary layer. Seasonal changes in water temperature and insolation also influenced δ^13^C.
3. δ^15^N values primarily reflected the δ^15^N values of DIN and varied along reef-scale pollution gradients. The relative influence of pollution-derived N on coral nutrition can be evaluated using the δ^15^N signature of host coral tissues or their symbiotic algae.
4. Heterotrophy by coral holobionts was shown to cause significant shifts in both δ^15^N and δ^13^C values. To use δ^15^N and δ^13^C values to evaluate nutrient sources and coral health, the dependence of coral holobionts on heterotrophy should be assessed and, if necessary, the effect of heterotrophy on δ^15^N and δ^13^C values should be adequately corrected, e.g., based on the compound-specific δ^15^N values of amino acids.

## Figures and Tables

**Table 1:** One-way ANOVA results for dependence of the C/N ratio, δ^15^N, and δ^13^C on season, species, and site (see also Figure 3 and Figure S1).

Parameter		Dependence on	
	Season	Species	Site
*C/N ratio*			
Algal symbiont	–	+++	+++
Host coral	++	+++	+++
Difference	++	+++	+++
*Bulk δ^15^N*			
Algal symbiont	+++	+++	+++
Host coral	+	++	+++
Difference	–	+++	+
*Bulk δ^13^C*			
Algal symbiont	–	+++	+
Host coral	–	+++	+++
Difference	+++	+	–

+++: *p* < 0.0001; ++: *p* < 0.001; +: *p* < 0.05; and –: not significant.

**Table 2:** Nutrient concentrations and isotopic compositions of representative freshwater sources near the study sites.

Sample	Most Affected Station	Season	NH_4_^+^		NO_3_^−^	
			Conc. (µM)	δ^15^N	Conc. (µM)	δ^15^N
River water	Station 4	June 2010	1.8	nd	120	7.5
		September 2010	<0.1	nd	170	7.6
		January 2012	0.7	nd	180	nd
Groundwater	Station 3	August 2010	<0.1	nd	300	8.8
	Station 9	June 2010	1.1	nd	380	6.4
		September 2010	<0.1	nd	310	6.2
		January 2012	0.1	nd	310	nd
Sewage water	Station 10	July 2005	110–550	21.2	9–72	nd

Nutrient concentrations were determined by an AutoAnalyzer (AACS II, BL-TEC, Osaka, Japan). The δ^15^N of ammonium was determined by the method of [81]. The δ^15^N of nitrate was determined by the denitrification method [82]. nd: not determined. Sewage water data are taken from [83]. The other data are the authors’ unpublished data.

**Table 3:** Comparison of the degree of nitrogen isotope fractionation of individual amino acids against Glx in the symbiotic algal fractions of coral samples.

Reference	“Non-Fractionating”				“Fractionating”							
	Asx	Ala	Val	Pro	Thr	Phe	Gly	Ile	Leu	Ser	Lys	Met
This study	−0.14 ± 1.15	−1.88 ± 1.53	−0.13 ± 1.93	(−4.4 ± 3.7) *	−2.21 ± 3.98	−3.74 ± 1.17	−4.43 ± 1.01	−2.98 ± 1.66	0.02 ± 1.97	−2.31 ± 1.45	nd	−3.82 ± 0.94
Sample number (*n*)	10	10	10	6	10	10	10	9	10	10	0	6
McCarthy et al. [91]												
Eukaryotic algae	−1.62 ± 1.34	−0.19 ± 1.08	0.35 ± 1.61	−0.77 ± 2.88	−3.02 ± 1.37	−1.39 ± 1.75	−8.50 ± 1.58	−3.86 ± 1.06	−6.72 ± 0.55	−3.34 ± 1.90	−6.30 ± 1.11	nd
Cyanobacteria	−0.16 ± 1.08	−0.49 ± 2.08	0.97 ± 1.62	0.97 ± 1.13	0.49 ± 1.59	−2.63 ± 1.54	−0.84 ± 1.77	−2.62 ± 0.96	−2.53 ± 1.28	−5.36 ± 0.85	−4.30 ± 2.04	nd

* Unreliable estimate due to bad chromatographic resolution of peaks. nd: no data.

**Table 4:** Correlation of Δδ^15^N_x–Phe_ (difference in δ^15^N of an individual amino acid (x) from that of phenylalanine) with Δδ^15^N_Glx–Phe_ in the host coral fraction of corals (n = 18).

Δδ^15^N_x–Phe_	Statistics		
x	Slope	Offset	*r*
Ala	1.19	−0.18	0.720 *
Gly	0.98	−5.25	0.637 *
Val	1.46	−1.63	0.802 **
Leu	1.78	−1.07	0.830 ***
Ile	1.94	−6.08	0.611 *
Pro	1.72	−9.49	0.587
Asx	0.86	1.24	0.841 ***
Thr	0.34	−2.09	0.221
Ser	0.83	−2.46	0.608 *
Met	0.07	−0.52	0.073

Slope (*a*) and offset (*b*) of linear regression model (Δδ^15^N_x–Phe_ = *a Δ*δ^15^N_Glx–Phe_ + *b*). Correlation was evaluated by ANOVA. ***: *p* < 0.005; **: *p* < 0.01; and *: *p* < 0.05.

## Supplementary Materials

The following are available online at https://www.mdpi.com/2076-2607/8/8/1221/s1, **Figure S1**. Differences in C/N atomic ratios (ΔC/N_a–h_; a, d, g), bulk δ^15^N values (Δδ^15^Na–h; b, e, and h), and bulk δ^13^C values (Δδ^13^C_a–h_; c, f, and i) between the algal symbiont and host coral fractions compared among seasons (a–c), species (d–f), and sampling sites (g–i). Differences among seasons, species, and sites were evaluated using the Tukey–Kramer multiple comparison test (α = 0.05); different characters at the top of the error bars indicate statistically significant differences. Lack of characters means no significant differences detected. **Table S1.** Description of sampling sites and numbers of coral samples analyzed. **Table S2**. Seasonal differences in C/N ratios, δ^13^C values, and δ^15^N values of algal symbionts and host corals evaluated via ANOVA with Bonferroni–Dunn post hoc analysis. **Table S3**. Species-specific differences in C/N ratios, δ^13^C values, and δ^15^N values of algal symbionts and host corals evaluated via ANOVA with Bonferroni–Dunn post hoc analysis. **Table S4**. Site-specific differences in C/N ratios, δ^13^C values, and δ^15^N values of algal symbionts and host corals evaluated via ANOVA with Bonferroni–Dunn post hoc analysis. **Table S5**. Amino acid compositions (mole-%) of the host coral and symbiotic algal fractions of coral holobionts (*n* = 10).
