# Supplementary material for: Organic Carbon and Nitrogen Isoscapes of Reef Corals and Algal Symbionts: Relative Influences of Environmental Gradients and Heterotrophy
Source: Microorganisms. 2020 Aug 11;8(8):1221. doi: 10.3390/microorganisms8081221 (PMC7464353; doi:10.3390/microorganisms8081221)
Supplement: Supplementary file 1 [file microorganisms-08-01221-s001.pdf]

microorganisms

Article

Organic carbon and nitrogen isoscapes of reef corals and algal symbionts: Relative influences of environmental gradients and heterotrophy

Supplementary materials

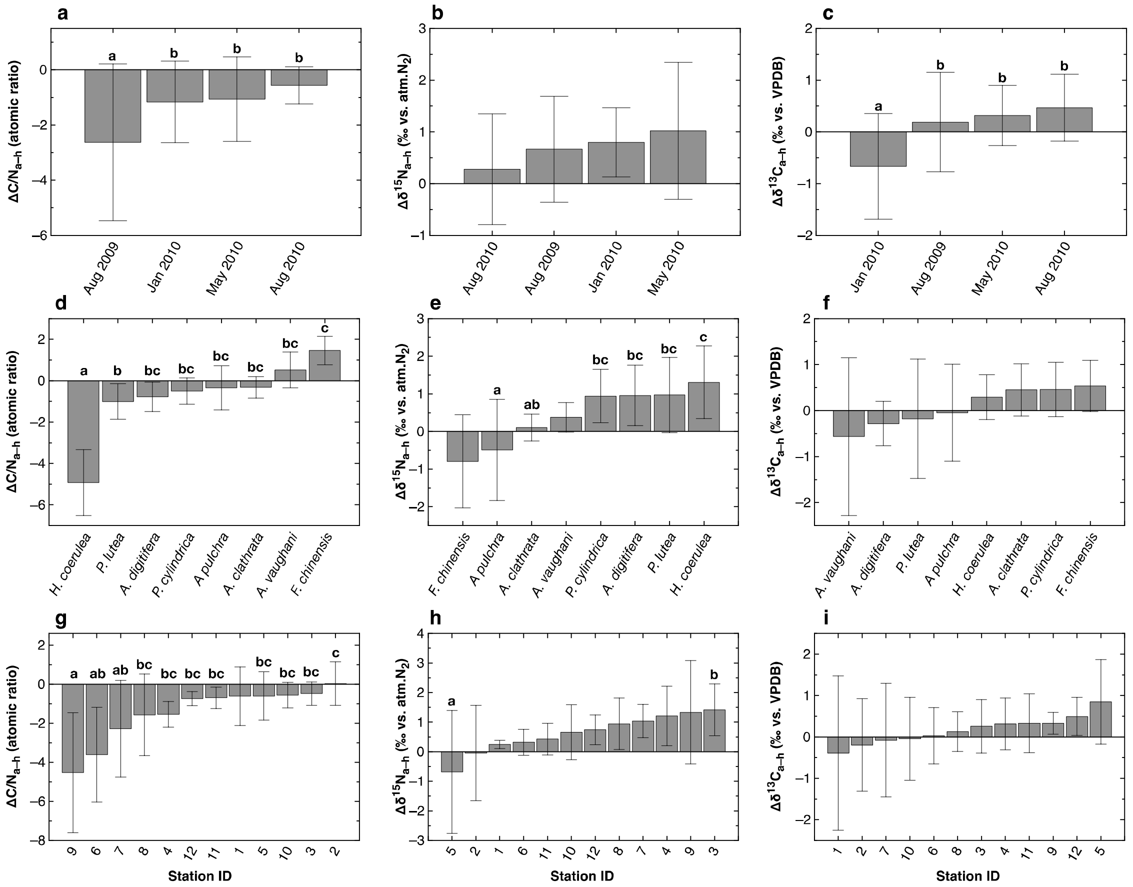

Figure S1. Differences in C/N atomic ratios ($\Delta C/N_{a-h}$; **a**, **d**, **g**), bulk $\delta^{15}N$ values ($\Delta\delta^{15}N_{a-h}$; **b**, **e**, **h**), and bulk $\delta^{13}C$ values ($\Delta\delta^{13}C_{a-h}$; **c**, **f**, **i**) between the algal symbiont and host coral fractions compared among seasons (**a**–**c**), species (**d**–**f**), and sampling sites (**g**–**i**). Differences among seasons, species, and sites were evaluated using the Tukey-Kramer multiple comparison test (α = 0.05); different characters at the top of the error bars indicate statistically significant differences. Lack of characters means no significant differences detected.

Table S1. Description of sampling sites and numbers of coral samples analyzed.

Site ID	Location		Water depth (m) at low tide		Number of coral samples*				
	Latitude (˚N)	Longitude (˚E)			Aug 2009	Jan 2010	May 2010	Aug 2010	Total
1	24.5950	124.3108	1.0	Shallow reef flat	3 (0)	0 (0)	0 (0)	0 (0)	3 (0)
2	24.5712	124.2982	1.0	Shallow reef flat	6 (0)	0 (0)	7 (0)	0 (0)	13 (0)
3	24.4231	124.2558	2.0	Near a big channel across the reef crest	0 (0)	0 (0)	8 (0)	0 (0)	8 (0)
4	24.3842	124.2553	2.0	Close to the Todoroki River mouth	5 (0)	0 (0)	4 (0)	0 (0)	9 (0)
5	24.3706	124.2551	2.0	Shallow reef flat	0 (0)	0 (0)	0 (0)	4 (0)	4 (0)
6	24.3652	124.2575	0.5	Close to the reef crest	10 (5)	0 (0)	0 (0)	0 (0)	10 (5)
7	24.3646	124.2552	1.0	Shallow reef flat	10 (5)	10 (3)	0 (0)	0 (0)	20 (8)
8	24.3652	124.2537	1.0	Shallow reef flat	0 (0)	8 (4)	14 (6)	0 (0)	22 (10)
9	24.3649	124.2510	0.5	Groundwater-affected area	8 (5)	0 (0)	0 (0)	0 (0)	8 (5)
10	24.3394	124.1989	1.0	Close to a sewage outfall	0 (0)	7 (0)	6 (0)	4 (0)	17 (0)
11	24.3410	124.0961	2.0	Inside Sekisei Lagoon	0 (0)	0 (0)	0 (0)	6 (0)	6 (0)
12	24.3484	123.9521	5.0	Inside Sekisei Lagoon	0 (0)	0 (0)	0 (0)	6 (0)	6 (0)

* Number in parenthesis indicates the sample number of *Heliopora coerulea*.

Table S2. Seasonal differences in C/N ratios, $\delta^{13}C$ values and $\delta^{15}N$ values of algal symbionts and host corals evaluated via ANOVA with Bonferroni–Dunn post-hoc analysis.

Coral species	Site ID	Seasons compared	Algal symbionts			Host corals		
			C/N	$\delta^{15}N$	$\delta^{13}C$	C/N	$\delta^{15}N$	$\delta^{13}C$
Acropora digitifera	10	Jan 2010 vs. May 2010 vs. Aug 2010	ns	ns	ns	– *	ns	ns
Acropora pulchra	2	Aug 2009 vs. May 2010	ns	ns	ns	Aug < May *	May < Aug **	May < Aug *
Heliopora coerulea	7	Aug 2009 vs. Jan 2010	ns	Jan < Aug ***	Jan < Aug ***	ns	ns	Jan < Aug **
Heliopora coerulea	8	Jan 2010 vs. May 2010	ns	ns	ns	ns	ns	ns
Porites cylindrica	8	Jan 2010 vs. May 2010	ns	ns	ns	ns	Jan < May *	ns
Porites lutea	4	Aug 2009 vs. May 2010	Aug < May *	ns	ns	ns	May < Aug **	Aug < May *
Porites lutea	10	Jan 2010 vs. May 2010	ns	ns	Jan < May **	ns	ns	ns

† Difference of means is shown only when it is statistically significant by ANOVA (*: $p < 0.05$; **: $p < 0.01$; ***: $p < 0.001$. ns: no significant difference was detected between seasons by ANOVA. –: no significant difference was detected by Bonferroni–Dunn post-hoc analysis.

Table S3. Species-specific differences in C/N ratios, δ^{13}C values, and δ^{15}N values of algal symbionts and host corals evaluated via ANOVA with Bonferroni–Dunn post-hoc analysis.

Station	Season	Species compared[§]	Algal symbionts			Host corals		
			C/N	δ^{15}N	δ^{13}C	C/N	δ^{15}N	δ^{13}C
2	Aug 2009	Ap vs. Av	ns	Ap < Av *	ns	Ap < Ac *	ns	Ap < Av *
2	May 2010	Ap vs. Pl	ns	ns	Ap < Pl *	ns	ns	Ap < Pl *
3	May 2010	Pc vs. Pl	ns	ns	ns	Pc < Pl ***	Pc < Pl *	Pc < Pl *
6	Aug 2009	Ad vs. Ap vs. Hc	ns	ns	Ad, Ap < Hc **	Ad, Ap < Hc **	Hc < Ad, Ap **	Ad, Ap < Hc **
7	Aug 2009	Hc vs. Pc	Pc < Hc **	Hc < Pc **	ns	Pc < Hc ***	Hc < Pc ***	ns
7	Jan 2010	Ap vs. Hc vs. Pl	Ap < Hc *	Ap, Hc < Pl **	Ap < Hc *	Ap, Pl < Hc ***	ns	Ap < Hc < Pl ***
8	Jan 2010	Hc vs. Pc	Pc < Hc ***	Hc < Pc ***	ns	Pc < Hc ***	ns	ns
8	May 2010	Fc vs. Hc vs. Pc	ns	ns	ns	Fc, Pc < Hc ***	Hc < Pc < Fc ***	ns
9	Aug 2009	Hc vs. Pc	Pc < Hc *	Hc < Pc *	ns	Pc < Hc **	Hc < Pc **	ns
10	Jan 2010	Ad vs. Pl	ns	ns	ns	Ad < Pl *	Ad < Pl *	Ad < Pl **
10	May 2010	Ad vs. Pl	Ad < Pl *	ns	Ad < Pl ***	ns	ns	Ad < Pl **
10	Aug 2010	Ac vs. Ad	ns	ns	ns	ns	ns	ns
11	Aug 2010	Ac vs. Ad	Ad < Ac **	ns	ns	ns	Ad < Ac *	ns
12	Aug 2010	Ac vs. Pc	Ac < Pc ****	Pc < Ac ***	Pc < Ac **	Ac < Pc ***	Pc < Ac ***	Pc < Ac ***

Difference of means is shown only when it is statistically significant by ANOVA (*: $p < 0.05$; **: $p < 0.01$; ***: $p < 0.001$. ns: no significant difference was detected between seasons by ANOVA. [§] Ac, *Acropora clathrata*; Ad, *A. digitifera*; Ap, *A. pulchra*; Av, *A. vaughani*; Fc, *Favites chinensis*; Hc, *Heliopora coerulea*; Pc, *Porites cylindrica*; Pl, *P. lutea*.

Table S4. Site-specific differences in C/N ratios, $\delta^{13}C$ values, and $\delta^{15}N$ values of algal symbionts and host corals evaluated via ANOVA with Bonferroni–Dunn post-hoc analysis.

Coral species	Season	Stations compared	Algal symbionts			Host corals		
			C/N	$\delta^{15}N$	$\delta^{13}C$	C/N	$\delta^{15}N$	$\delta^{13}C$
Acropora clathrata	Aug 2010	10 vs. 11 vs. 12	ns	11, 12 < 10 ***	ns	ns	11, 12 < 10 ***	ns
Acropora digitifera	Aug 2010	10 vs. 11	11 < 10 *	11 < 10 **	ns	ns	11 < 10 **	ns
Acropora pulchra	Aug 2009	1 vs. 2 vs. 6	ns	2, 6 < 1 ***	ns	ns	6 < 1 **	ns
Heliopora coerulea	Aug 2009	6 vs. 7 vs. 9	ns	6, 7 < 9 ***	ns	ns	7 < 9 *	ns
Heliopora coerulea	Jan 2010	7 vs. 8	ns	7 < 8 ***	ns	ns	7 < 8 **	7 < 8 **
Porites cylindrica	Aug 2009	7 vs. 9	9 < 7 *	7 < 9 ***	ns	ns	7 < 9 ***	ns
Porites cylindrica	May 2010	3 vs. 8	ns	3 < 8 *	ns	ns	3 < 8 ***	3 < 8 *
Porites lutea	Jan 2010	7 vs. 10	ns	7 < 10 ***	ns	ns	7 < 10 ***	ns
Porites lutea	May 2010	2 vs. 3 vs. 4 vs. 10	ns	– *	2, 4 < 10 *	10 < 2 *	2, 3 < 4 < 10 ***	ns

Difference of means is shown only when it is statistically significant by ANOVA (*: $p < 0.05$; **: $p < 0.01$; ***: $p < 0.001$. ns: no significant difference was detected between seasons by ANOVA. –: no significant difference was detected by Bonferroni–Dunn post-hoc analysis.

Table S5. Amino acid compositions (mole-%) of the host coral and symbiotic algal fractions of coral holobionts (n = 10).

	Ala	Gly	Val	Leu	Ile	Asx	Thr	Ser	Met	Glx	Phe	His	Tyr	Arg	Lys
This study															
– Host coral	6.8 ± 0.6	10.7 ± 1.7	6.7 ± 0.7	7.0 ± 0.9	4.6 ± 0.6	12.3 ± 3.2	5.5 ± 1.6	7.9 ± 1.8	0.0 ± 0.0	23.9 ± 5.9	3.8 ± 1.3	1.4 ± 0.6	0.5 ± 0.8	4.1 ± 0.7	4.2 ± 1.3
– Algal symbiont	8.4 ± 0.3	12.0 ± 1.3	7.5 ± 0.4	8.5 ± 0.8	5.3 ± 0.5	12.1 ± 1.3	6.1 ± 0.3	7.0 ± 0.4	0.1 ± 0.2	18.0 ± 3.7	3.7 ± 0.7	1.7 ± 0.4	1.2 ± 0.9	5.4 ± 0.9	3.9 ± 1.0
Fitzgerald and Szmant (1997) [72]															
– Host coral*	7 – 8	8 – 11	7 – 8	7 – 10	5 – 6	11 – 13	6 – 7	7 – 8	1 – 2	13 – 15	4 – 5	3 – 4	2 – 5	4 – 6	5 – 7

* Protein samples precipitated by trichloroacetic acid from suspension of host coral tissue.

© 2020 by the author. Licensee MDPI, Basel, Switzerland. This article is an open access article distributed under the terms and conditions of the Creative Commons Attribution (CC BY) license (http://creativecommons.org/licenses/by/4.0/).
